# Surgical management and outcomes of pure sacroiliac joint dislocations: A systematic review

**DOI:** 10.1016/j.jor.2025.01.005

**Published:** 2025-01-03

**Authors:** Salvatore Pantè, Marco Bufalo, Alessandro Aprato, Michele Nardi, Riccardo Giai Via, Francesco Bosco, Luca Rollero, Alessandro Massè

**Affiliations:** a University of Turin, Centro Traumatologico Ortopedico (CTO), Department of Orthopaedic Surgery, Turin, Italy; b University of Turin, Ospedale Infantile Regina Margherita, Department of Pediatric Orthopaedic Surgery, Turin, Italy; c Department of Precision Medicine in Medical, Surgical and Critical Care (Me.Pre.C.C.), University of Palermo, Palermo, Italy; d Department of Orthopaedics and Traumatology, G.F. Ingrassia Hospital Unit, ASP 6, Palermo, Italy

**Keywords:** Pelvic, Sacroiliac, Trauma, Injury, Spinopelvic

## Abstract

**Introduction:**

Sacroiliac joint (SIJ) dislocations, particularly pure SIJ dislocations without associated fractures, represent a rare and complex subset of pelvic ring injuries. Given the intricate pelvic anatomy and the need to achieve both stability and functional recovery, the optimal surgical management for these injuries remains a topic of debate. This systematic review aims to evaluate the various surgical techniques employed in treating this rare and challenging injury and assess associated clinical outcomes and complications.

**Materials and methods:**

A systematic review was conducted adhering to the PRISMA guidelines. Relevant studies were searched in four databases: Pubmed, Scopus, Embase, and Medline. The selected articles were evaluated according to the criteria of levels of evidence. The included studies were analyzed using the Methodological index for non-randomized studies (MINORS) criteria for quality assessment. This paper was registered in the International Prospective Registry of Systematic Reviews (PROSPERO).

**Results:**

The review identified four studies. The surgical techniques varied across studies, with percutaneous fixation, open reduction and internal fixation (ORIF), and combined approaches being the most reported methods. Clinical outcomes generally indicated satisfactory pain relief and functional recovery (Majeed score 57–90), though the rates of complications, including hardware failure (4–17 %) and infection (17–32 %), were notable. The results also highlighted that anatomical reduction and stable fixation are crucial for optimizing outcomes and minimizing complications. However, the heterogeneity of the data, especially the timing of surgery and surgical technique, precluded a formal meta-analysis.

**Conclusions:**

Surgical management of pure SIJ dislocations presents significant challenges due to the complex biomechanics of the pelvic ring. While various surgical techniques have been employed with generally positive outcomes, the lack of high-quality, large-scale studies limits the ability to establish definitive guidelines. Early definitive treatment of these injuries and anatomical reduction of the SIJ are the main factors to improve clinical outcomes and reduce complication rates.

**Level of evidence:**

IV.

## 1 Introduction

Pelvic ring disruptions are relatively uncommon injuries, with a prevalence of nearly 2–37 per 100.000 in the general population, and typically result from high-energy trauma.^1^ Such injuries have a wide range of clinical implications, are challenging to treat, and, although appropriate management, significantly impact patient's quality of life.^2^

Pure sacroiliac joint (SIJ) dislocations are the most challenging situations in terms of treatment and functional outcomes, as they are often associated with an unstable pelvic ring due to complete ligamentous disruption leading to misalignment of the sacrum and the iliac bone.^3^ If these injuries are not treated appropriately, patients may refer symptoms of chronic pelvic ring instability, deformities, and functional disorders, such as leg length discrepancy, gait abnormalities, sitting problems, and low back pain. Achieving anatomical reduction and stable fixation is crucial.^4^^,^^5^

Results of short-term and mid-term follow-up studies after pelvis fracture underline the need for excellent reduction.^6^ Rapid fixation and early mobilization are now the gold standard. Still, most of these injuries are the consequence of polytrauma, and these patients may not be fit for surgery at the time of arrival in the emergency department. If necessary, damage control surgery with an external fixator aims to control the bleeding and early stabilize the fractures.^7^ The external fixator may not be enough to stabilize the pelvis, and advanced techniques, such as the antishock iliosacral screw, help to achieve good clinical and mechanical stability.^8^ The timing of surgery is difficult to predict precisely in these situations, and the delay might be due to the patient's clinical condition or referral to high-volume hospitals with experienced surgeons. This must be considered in the preoperative planning, especially for the decision of open or closed reduction of the dislocation. Various methods of fixation are available, from percutaneous fixation to open intrapelvic surgery and spinopelvic fixation, and each method has its advantages and disadvantages that should be considered case-by-case. The most reported technique to stabilize pelvic ring injuries is iliosacral screw fixation, which is performed percutaneously after closed reduction of the dislocation, allowing to achieve good compression perpendicularly to the iliosacral joints with less invasivity and soft tissue dissection.^9^^,^^10^ However, it's still not clear which is the best method to achieve a stable fixation in pure ligamentous SIJ dislocations.

Pure SIJ dislocations are poorly reported in the literature, and most studies on this topic report posterior ring injuries without distinguishing between crescent fracture-dislocations, sacral fractures, and pure sacroiliac dislocations. This type of pelvic injury has historically been a more significant injury with a higher likelihood of poor functional outcomes than posterior ring fracture or fracture-dislocation.^11^

This systematic review aims to critically analyze the clinical outcomes of pure sacroiliac joint (SIJ) dislocations, focusing exclusively on cases without associated fractures, treated with various surgical techniques. By synthesizing current evidence, the review seeks to identify patterns in clinical outcomes, assess complication rates, and provide insights into the efficacy of different fixation methods to guide clinical decision-making in this rare and complex injury. We hypothesize that anatomical reduction and stable fixation of pure SIJ dislocations significantly improve functional outcomes and minimize complications, regardless of the specific surgical technique employed.

## 2 Materials and methods

### 2.1 Research question

**Fig. 1:**
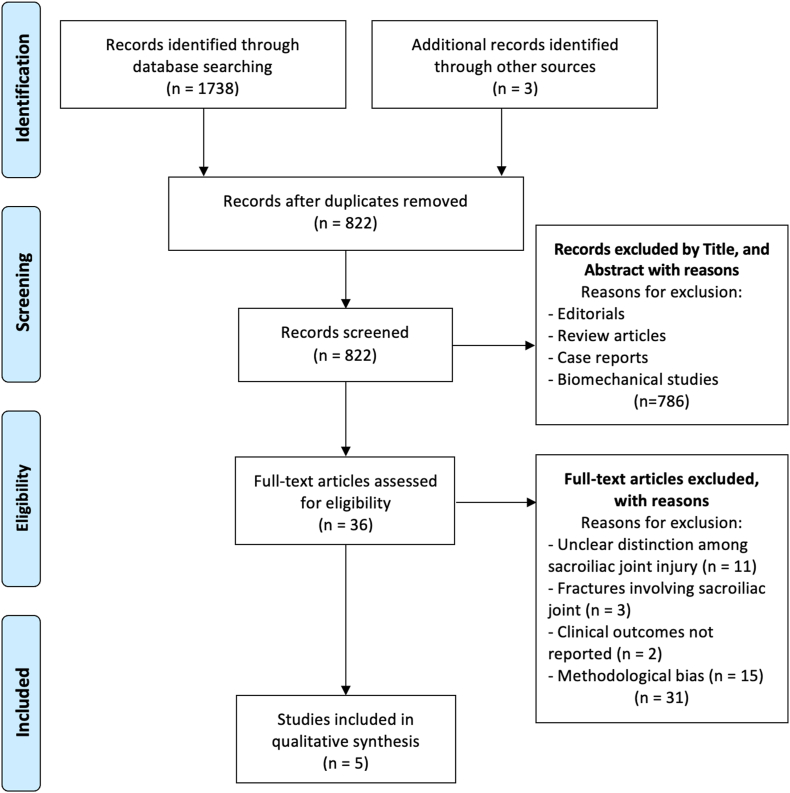
Prisma flow diagram.

This study adhered to the Preferred Reporting Items for Systematic Reviews and Meta-Analyses (PRISMA) guidelines (Fig. 1).^12^ Two authors (SP and MB) independently searched and evaluated the articles to avoid bias. In cases of uncertainty, a senior author (LR) was consulted to resolve doubts.

### 2.2 Inclusion and exclusion criteria

The inclusion criteria for the selected studies required articles focusing on patients who underwent surgical treatment for sacroiliac dislocations without associated fractures involving the joint. Eligible studies had to be written in English, conducted on human subjects, published between January 2000 and June 2024, and report a minimum mean follow-up of 12 months. Only randomized controlled trials (RCTs), prospective studies, and retrospective studies with Levels of Evidence (LoE) 1 to 4 were included.^13^ Exclusion criteria encompassed biomechanical research, case reports, editorials, book chapters, technical notes, pre-clinical investigations, review articles, and studies that did not clearly differentiate sacroiliac joint dislocations from fractures involving the joint or failed to report clinical outcomes.

### 2.3 Search strategy and study selection

A comprehensive literature search was conducted across four databases—PubMed, Scopus, Embase, and Medline—using the following MeSH terms: ((posterior pelvic ring) OR (sacroiliac dislocation)) AND ((outcome) OR (fixation)). The search focused on studies published between January 2000 and June 2024. Concluding on June 11, the search identified a total of 1738 studies. Following the removal of duplicates, 822 studies remained for initial screening. After applying the inclusion and exclusion criteria, 36 studies were deemed eligible. A detailed full-text evaluation further narrowed this down to five studies, which were selected for qualitative and quantitative analysis. The studies included in the review provided data on patient injuries, surgical techniques, functional outcomes, and complication rates.

### 2.4 Methodological quality assessment

The selected articles were categorized according to the Oxford Centre for Evidence-Based Medicine 2011 Levels of Evidence (LoE), which ranks studies from 1 to 5. LoE 1 indicates superior design, higher methodological quality, and minimal risk of bias. The Methodological Index for Non-Randomized Studies (MINORS) criteria was applied to assess the quality of the included studies^14^ (Table 1). Two authors (SP and MB) independently performed the quality assessment, with a third author (MN) consulted to address any disagreements or uncertainties. This systematic review was registered in the International Prospective Register of Systematic Reviews (PROSPERO) under the registration number CRD42024570931 in July 2024.^15^

**Table 1:** Qualitative analysis of included studies using the Methodological Index for Non-Randomized Studies (MINORS) criteria, detailing the evaluation of methodological quality across various domains.

Authors	YoP	MINORS	A clearly stated aim	Inclusion of consecutive patients	Prospective collection of data	Endpoint appropriate for aim of study	Unbiased assessment of study endpoint	Follow-up period appropriate	Loss of follow-up less than 5 %	Prospective calculation of study size
Abumi et al.^16^	2000	12	2	2	2	2	0	2	2	0
Mullis et al.^17^	2008	11	2	2	2	2	0	1	2	0
Sobhan et al.^18^	2016	12	2	2	2	2	0	2	2	0
Gauthè et al.^19^	2022	12	2	2	2	2	0	2	2	0
Miyake et al.^20^	2022	12	2	2	2	2	0	2	2	0

YoP = Year of publication; MINORS = Methodological index for non-randomized studies.

### 2.5 Data extraction

Data from the selected studies were systematically extracted and recorded using Excel spreadsheets by two authors working independently (SP and MB). Once completed, the data sets were merged for consistency. The extracted information included the following key elements: author and publication year, study design, patient sample size, mean age, gender distribution, side of injury, mean follow-up duration, surgical techniques utilized, complication rates, and functional outcomes. This approach ensured a structured and comprehensive analysis of the findings. A third author (RGV) reviewed the entire manuscript upon completion of the draft to ensure accuracy and coherence.

### 2.6 Statistical analysis

Statistical analysis was carried out using R software (version 4.1.3, R Core Team, Vienna, Austria). Descriptive statistics were employed to summarize the data extracted from the included studies. Measures of variability were reported as standard deviations (SD) or as ranges between the minimum and maximum values. For categorical variables, absolute frequencies and relative distributions were calculated, providing a detailed and accurate representation of the data for further interpretation.

## 3 Results

The systematic search and selection process identified five studies^16^, ^17^, ^18^, ^19^, ^20^ specifically addressing the surgical management of pure SIJ dislocations. These studies included a total of 62 patients, 4 of whom presented with bilateral injuries, and 13 were female. Two of the studies reported various types of pelvic injuries but provided sufficient detail to enable the inclusion of only those cases involving pure SIJ dislocations.

All included patients underwent surgical treatment, which was characterized by significant variability in techniques and types of implants used, reflecting the diversity of approaches employed in managing these complex injuries.

### 3.1 Trauma assessment

The mechanism of injury was detailed in three studies,^16^^,^^18^^,^^19^ identifying motor vehicle accidents and falls from significant heights as the primary causes (Table 2). Sacroiliac joint (SIJ) dislocation was frequently associated with other pelvic injuries, most commonly involving the pubic symphysis and the superior and inferior pubic rami.

**Table 2:** Summary of included studies and patient characteristics.

Authors	YoP	Study design	Total number of patients, N	Age, years, Mean (range Min-Max)	Male, N	Female, N	Side (U/B), N/N	Cause of injury, (N)
Abumi et al.^16^	2000	Retrospective	7	39.4 (22–55)	5	2	7/0	- Motor vehicle accident^4^ - Industrial injury^3^
Mullis et al.^17^	2008	Retrospective	23	/	/	/	/	/
Sobhan et al.^18^	2016	Retrospective	6	38.7 (24–70)	5	1	5/1	- Accident^5^ - Falling^1^
Gauthè et al.^19^	2022	Retrospective	22	37.3 (20.6–62.2)	13	9	19/3	- Motor vehicle accident^12^ - Falling^10^
Miyake et al.^20^	2022	Retrospective	4	42 (20–67)	3	1	4/0	/

YoP = year of publication; N = Number; Min = minimum; Max = maximum;/= not specified; U/B = unilateral/bilateral.

Neurovascular injuries were noted in three studies,^16^^,^^18^^,^^19^ although it remains unclear whether these injuries were directly attributable to the SIJ dislocation itself. Additional associated injuries were documented in four studies.^16^^,^^18^, ^19^, ^20^

The time interval between trauma and surgical intervention was reported in three studies,^16^^,^^18^^,^^19^ demonstrating considerable variability. This variability likely reflects the impact of concurrent injuries and the complexities of clinical management for these patients (Table 3).

**Table 3:** Trauma assessment, associated injuries and time from injury to surgery.

Authors	YoP	Associated pelvic lesions, (N)	Neuro-vascular injuries, (N)	Associated injuries, (N)	Days from injury to surgery, Mean (range Min-Max)
Abumi et al.^16^	2000	- Disruption of the symphysis pubis^4^ - Fracture of superior and inferior rami^3^ - Sacral fracture^1^	Femoral and sciatic nerve palsy on the ipsilateral side^1^	- Lung and liver injury^1^ - Bladder rupture^1^	13^8^, ^9^, ^10^, ^11^, ^12^, ^13^, ^14^, ^15^, ^16^, ^17^, ^18^, ^19^, ^20^, ^21^, ^22^
Mullis et al.^17^	2008	/	/	/	/
Sobhan et al.^18^	2016	- Bilateral acetabular fracture and ramus pubis fracture^1^	Sciatic nerve palsy^1^	Liver rupture^1^	39 (6–180)
		- Pubic dislocation^3^			
		- Pubic fracture^1^			
Gauthè et al.^19^	2022	/	Neurological deficits^11^	- Thoracic, digestive, brain 15	7.2^1^, ^2^, ^3^, ^4^, ^5^, ^6^, ^7^, ^8^, ^9^, ^10^, ^11^, ^12^, ^13^, ^14^, ^15^, ^16^, ^17^, ^18^, ^19^, ^20^, ^21^, ^22^, ^23^, ^24^, ^25^, ^26^, ^27^, ^28^, ^29^, ^30^, ^31^, ^32^, ^33^, ^34^, ^35^, ^36^
				- Rectal^2^	
				- Urological^11^	
Miyake et al.^20^	2022	Hip dislocation^1^	/	- Liver injury^1^	/
				- Multiple rib fractures^2^	

YoP = year of publication; N = Number; Min = minimum; Max = maximum;/= not specified.

### 3.2 Surgical technique

The surgical techniques described in the included studies demonstrated considerable variability but adhered to the common principle of achieving anatomical reduction and stable fixation of the SIJ dislocation (Table 4). Abumi et al.^16^ reported the use of a combined technique involving S1 pedicle screws and the Galveston method, which facilitated direct reduction of the SIJ dislocation and stabilization of both the iliac wing and sacrum. Mullis et al.^17^ was the only study to describe the application of sacroiliac screws, inserted after either closed or open reduction, specifically for pure SIJ dislocations. Sobhan et al.^18^ implemented spinopelvic fixation, which included constructs made up of pedicle screws, iliac screws, and connecting rods to maintain stability. Gauthè et al.^19^ introduced a posterior hinge fixation method that allowed horizontal motion of the SIJ, utilizing a combination of screws and a rod that was not locked to the connectors. Miyake et al.^20^ described an innovative anterior approach to the SIJ using spinal instrumentation through the first window of the ilioinguinal approach, where polyaxial screws were placed into the lateral sacrum and medial iliac bone and connected with a rod.

**Table 4:** Surgical treatment and postoperative indications.

Authors	YoP	Surgical technique	Additional anterior pelvic ring fixation, (N)	Postoperative indications
Abumi et al.^16^	2000	S1 pedicle screws and Galveston technique	Symphysis plate^3^	- Sitting within 7 days
				- Walking after 3–6 weeks, depending on injury type
Mullis et al.^17^	2008	Single iliosacral screw (7.3 mm with 32 mm thread)	Symphysis plate^23^	- Early mobilizations after 24h
				- No weight-bearing for 3 months
Sobhan et al.^18^	2016	Spinopelvic fixation	/	Partial weight bearing after surgery and full weight bearing after complete union of the injury
Gauthè et al.^19^	2022	Posterior hinge fixation	Symphysis plate^7^	No weight bearing for 6 weeks and then progressive return to full weight-bearing
Miyake et al.^20^	2022	Anterior sacroiliac stabilization	Symphysis plate^2^	/

YoP = year of publication; N = Number;/= not specified; mm = millimeter.

In all cases, posterior fixation was reinforced with symphysis plating when there was a significant concomitant anterior injury, providing additional stability to the pelvic ring. Postoperative protocols varied widely across the studies, particularly regarding weight-bearing recommendations, which appeared to depend on the stability provided by the posterior fixation and the presence of anterior reinforcement. These diverse techniques reflect surgical management's complexity and individualized nature for this challenging pathology.

### 3.3 Reported outcomes

All the included studies provided data on the clinical outcomes of patients following surgical treatment (Table 5). Four studies^17^, ^18^, ^19^, ^20^ utilized standardized scoring systems, such as the Majeed score, to evaluate functional recovery and quality of life. In contrast, one study^16^ assessed outcomes based on pain levels, gait functionality, and the ability to return to work.

**Table 5:** Clinical outcomes and complications.

Authors	YoP	Outcome measure, Mean (range Min-Max)	Reduction quality, (N)	Clinical follow-up, months, Mean (range Min-Max)	Complications, (N)
Abumi et al.^16^	2000	Pain: none in 5 patients	Vertical displacement <5 mm^5^	43 (25–70)	Deep infection^1^
		Gait: normal in 6 patients			
		Employment: 5 patients returned to their jobs			
Mullis et al.^17^	2008	11 patients with malunion at 1 year: - Majeed score: 57 - Iowa score: 62 - SF-36: 90 - MFA: 208 12 patients with anatomic reduction at 1 year: - Majeed score: 77 - Iowa score: 83 - SF-36: 93 - MFA: 114	Vertical displacement <5 mm^12^	(13–120)	/
Sobhan et al.^18^	2016	Majeed score: 73.16 ± 16.88	Vertical displacement <5 mm^6^	27.6 (6–56)	- Infection^1^
					- Hardware failure^1^
Gauthè et al.^19^	2022	- Majeed score: 76.4 ± 15.3 (36–100) - VAS: 28 ± 23 (0–60)	/	57.6 (12–138)	- Surgical site infection^7^
					- Pulmonary embolism^1^
					- Lower leg ischemia^1^
					- Nonunion^12^
					- Mechanical failure^1^
Miyake et al.^20^	2022	Majeed score: 90 (85–96)	/	26 (7–45)	/

YoP = year of publication; N = Number;/= not specified; Min = minimum; Max = maximum; mm = millimeter; SF-36 = Short Form Health Survey-36 questions; MFA = Musculoskeletal Function Assessment; VAS = Visual Analogue Scale.

The follow-up duration varied significantly across the studies, but the mean follow-up time in all cases exceeded 12 months, allowing for a thorough evaluation of both short- and mid-term outcomes. These findings provide valuable insights into the effectiveness of different surgical approaches for managing pure SIJ dislocations.

### 3.4 Reduction of quality and complications

The quality of reduction, specifically measured by vertical displacement, was assessed in three studies.^16^, ^17^, ^18^ A displacement of less than 5 mm was considered indicative of anatomical reduction, highlighting the importance of precise alignment in achieving optimal outcomes.

Complication rates and types were documented in four studies.^16^, ^17^, ^18^, ^19^ Among the reported complications, nonunion of the dislocation was the most frequent. These findings, along with other complications, are detailed in Table 5, providing a comprehensive overview of the challenges encountered in managing this injury.

## 4 Discussion

The most significant finding of this systematic review is that effective surgical management involving proper reduction and stable fixation of SIJ dislocations leads to favorable postoperative clinical outcomes. Pure SIJ dislocations should be regarded as a distinct injury category due to the involvement of anterior and posterior sacroiliac ligaments. Unlike bone fractures, where healing is primarily based on bone regeneration, ligamentous injuries require a very stable fixation and generally demand longer healing times.^21^^,^^22^

The literature on pelvic ring fractures presents significant heterogeneity, with studies often combining various injury types, treatment modalities, and outcome measures.^21^^,^^22^ This lack of standardization complicates the evaluation and comparison of data.^23^ Furthermore, most studies fail to differentiate clinical and radiological outcomes based on specific injury patterns.^21^, ^22^, ^23^ While sacral fractures and sacroiliac fracture-dislocations reportedly achieve satisfactory outcomes with sacroiliac screws,^24^ the outcomes for pure SIJ dislocations without associated posterior ring fractures remain less clear.

This systematic review analyzed five distinct surgical techniques for managing pure SIJ dislocations. These ranged from single iliosacral screw fixation to more robust approaches, such as spinopelvic fixation or sacroiliac stabilization. All methods achieved acceptable good clinical outcomes for patients post-surgery.

However, several important considerations must be addressed. Variability in surgical techniques and postoperative protocols, the distinct healing mechanisms of ligamentous structures, and the limited number of high-quality studies focused exclusively on pure SIJ dislocations highlight the need for further research to establish standardized guidelines and improve clinical outcomes.

Abumi et al.^16^ reported the use of the Galveston technique combined with S1 pedicle screws to approach SIJ dislocations. This is interesting because it allows the surgeon to restore the posterior biomechanics of the pelvis and SIJ, especially without involving the lumbosacral spine and altering its physiological motion. This construct has a strong link with the anatomical reduction of the SIJ that must be achieved before the final fixation and the open posterior approach helps achieve this objective. However, we must consider that these injuries are usually associated with a disruption of the anterior pelvic ring, so this construct wouldn't be rigid enough for SIJ healing in the long term. In the paper, all seven patients had an anterior ring disruption, but only three underwent fixation of the symphysis with a DC plate, while one symphysis disruption and all pubic rami fractures were not fixed. This was explained for the possible complications of the ilioinguinal approach to plate these fractures. Still, nowadays, there are other possible alternatives to this, such as percutaneous screw fixation of pubic rami fracture^25^ or temporary external or internal fixation^16^ to improve the stability of the pelvis.

Mullis et al.^17^ treated these injuries with open reduction and internal fixation of the symphysis diastasis first and then closed or open reduction of SIJ dislocation and further fixation with a single iliosacral screw. This paper reports the most diffuse mean of fixation for this type of injury and focuses on the quality of reduction. The iliosacral screw is the most technical reproducible, cost-effective, less invasive, and time-saving technique among the ones reported in this systematic review. It has a lot of benefits, but the most important thing is to achieve a good quality of reduction before percutaneous fixation. The main problem with this approach is the technical difficulty of achieving a good closed reduction of SIJ; in fact, the authors report that open reduction must be taken into account if this is not possible with external maneuvers.^26^ Nowadays, there is no consensus on the timing for treating this type of injury. Still, the excessive delay of definitive surgery due to referral in specific centers or patient's comorbidities can lead to an extensive callus or fibrotic tissue, making close reduction extremely difficult or at least impossible.^27^^,^^28^ As proposed for percutaneous treatment of other pelvic injuries, the definitive fixation after closed reduction should be achieved as soon as possible, in emergency treatment, or in the first 48 h after temporary stabilization.^29^ Open reduction should be the first choice to achieve anatomical reduction if the definitive surgery is postponed for more than 7 days.^30^ Delayed surgery is associated with higher complication rates, lower reduction quality, and poorer short- and long-term outcomes.^28^ Anterior plating after anatomical reduction helps to restore a good SIJ alignment but it is not always enough. Another issue with this technique is due to the use of just a single screw to stabilize the SIJ. The anterior plating increases the stability of the pelvic ring, but a single iliosacral screw would allow micromotion of the joint, leading to secondary displacement^31^ and further nonunion or malunion of the SIJ dislocation with poorer clinical outcomes, about 50 % of the cases in Mullis et al. paper.^17^ The use of multiple iliosacral screws or trans-sacral screws would increase pelvic stability and reduce secondary displacement.^32^

Sobhan et al.^18^ reported the use of spinopelvic fixation in different combinations, depending on the specific posterior injury. This construct provides good stability after reduction and fixation, and it can also be used as a means of reduction after screws positioning in the iliac bone and the sacrum (S1 or S2). The use of an L5 pedicle screw reinforces the construct stability to contrast the vertical displacement of these types of injuries. This procedure has some difficulties, especially because it requires the patient to be in a prone position, making the reduction more difficult without directly looking at the anterior portion of the SIJ, which is the main reference for quality reduction in the axial and coronal plane.^33^ Wound infection and hardware intolerance are also concerns for this technique. Wound infection and dehiscence are more frequently reported with this approach for the continuous pressure on the lower back due to the bed rest of these patients and also because soft tissues of this body part have a poorer blood supply and are often involved in the initial trauma.^34^ However, there has been an increasing interest in minimally invasive spine surgery in recent years. This allowed to development of new percutaneous fixation systems, reducing blood loss, soft tissue stripping, and infection rate compared to open surgery.^35^ Regarding hardware management, it's well-known the discomfort often reported by the patient due to instrumentation prominence and, for this reason, these patients need to undergo surgery a second time for removal. But they are not the only ones because this procedure changes the lumbosacral spine biomechanics, leading to vertebral disc suffering and chronic back pain, so after about one year since fixation and after confirmation of healing, the hardware needs to be removed.^36^ In selected cases, avoiding the use of the L5 pedicle screw would allow the patients to preserve their spine biomechanics and further surgeries.

Gauthè et al.^19^ adopted a posterior hinge fixation, which has a biomechanical principle similar to the Galveston technique but differs for the implant and the sacral fixation. Lumbosacral fixation is not mandatory but can be evaluated case by case if there is a relevant vertical displacement or instability. The anterior symphysis fixation followed the posterior fixation every time there was no contraindication, and due to the presence of suprapubic cystostomy, rectal wounds, or external fixation pin wound suspect infection, it was done only in 32 % of the cases in this paper. One more time, anterior fixation is necessary for this type of injury to increase pelvic ring stability. Two interesting data are about surgical wound infection and nonunion, with higher rates compared to the other papers included in this systematic review. The early wound infection rate reflects what was reported previously on the poor blood supply of this area and the risk of pressure ulcers, in addition to the proximity of the anorectal region. Also, the nonunion rate was very high, and the possible cause was not clearly reported; it would be interesting to evaluate if the symphysis plating is related to a higher posterior ring injury union rate.

Miyake et al.^20^ readapted spinal instrumentation to fix posterior ring injuries through an anterior intrapelvic approach. This novel technique has some benefits for the surgeon: good anterior exposure of the SI joint, allowing direct visualization of the articular borders and the reduction quality, and use of versatile implants, helping the surgeon to achieve good reduction, compression and distraction of the joint, and angular stability after fixation. However, we must consider that the anterior approach to the SIJ is very difficult, and it requires deep tissue stripping to gain good exposure and to implant the screws in the proper position, with a high risk of complications like nerve and vascular injuries. It is not clear if the implants need to be removed after injury healing, especially because SIJ fusion is not achievable with this procedure without cartilage removal from the joint. So, in the long-term follow-up, some of the implants may break.

Each surgical technique employed for SIJ dislocations has its own advantages, disadvantages, learning curve, and specific indications. The selection of an appropriate technique depends on the individual patient's injury pattern, the surgeon's expertise, and the available resources. However, regardless of the technique used, achieving precise anatomical reduction and stable fixation remains the cornerstone of successful management. These two factors are critical prognostic indicators for healing SIJ dislocations and are directly linked to favorable clinical outcomes.^37^^,^^38^ The challenge lies in balancing the technical demands of these procedures with the goal of restoring pelvic stability and function while minimizing complications and long-term morbidity.

This study has several limitations that must be acknowledged. First, the number of included studies is small, and they are primarily retrospective, with limited sample sizes. This inherently restricts the generalizability of the findings. Second, the heterogeneity of patient groups, selection criteria, postoperative rehabilitation programs, and follow-up protocols across the studies complicates direct comparisons and reduces the reliability of pooled conclusions. Third, varied reduction and fixation techniques further contribute to the lack of standardization. Additionally, follow-up periods were highly variable, ranging from 6 to 138 months, which could introduce bias in assessing long-term outcomes. A more uniform and standardized follow-up approach would enhance the validity and comparability of the data. Another critical limitation is the insufficiently detailed descriptions of injury patterns in some studies, particularly regarding distinctions between pure SIJ dislocations and nearby fractures, such as sacral or crescent fractures. Moreover, none of the included studies reported data on chronic low back pain, a potential indicator of poor SIJ reduction, nonunion, or malunion. Chronic low back pain can necessitate secondary or revision surgeries, especially in cases with suboptimal fixation.^39^

In this complex clinical context, determining the precise source of lower back pain poses significant challenges. Patients may present with overlapping symptoms stemming from spine or nerve injuries, which can mimic those caused by SIJ dislocations. This diagnostic ambiguity complicates both the management and interpretation of outcomes, emphasizing the need for comprehensive and detailed reporting in future studies.

## 5 Conclusions

Sacroiliac joint dislocation is a severe and debilitating pelvic injury often characterized by multiplanar instability and frequently associated with multiorgan trauma due to high-energy mechanisms. Despite the critical nature of these injuries, there remains no consensus on the optimal management strategy. Various surgical techniques have been described, each with its advantages and limitations. Regardless of the method used, the critical determinants of favorable long-term clinical outcomes are minimizing delays to definitive treatment and achieving accurate anatomical reduction. These factors are pivotal in improving functional recovery and reducing the risk of complications. Future research should focus on developing standardized guidelines and treatment protocols to enhance patient care in this complex clinical scenario.

## CRediT authorship contribution statement

**Salvatore Pantè:** Conceptualization, Writing – original draft, preparation. **Marco Bufalo:** Writing – original draft, preparation, Methodology. **Alessandro Aprato:** Visualization, Supervision. **Michele Nardi:** Writing – original draft, preparation. **Riccardo Giai Via:** Investigation, Visualization. **Francesco Bosco:** Visualization, Supervision. **Luca Rollero:** Supervision. **Alessandro Massè:** Supervision.

## Guardian/patient's consent

Not Applicable.

## Ethical statement

The study was conducted following the ethical standards of the Declaration of Helsinki (1964).

## Funding statement

This research did not involve any specific grants from commercial, public, or non-profit sector funding agencies.

## References

[1] Pereira G.J.C., Damasceno E.R., Dinhane D.I., Bueno F.M., Leite J.B.R., Ancheschi B.D.C. (2017). Epidemiology of pelvic ring fractures and injuries. Rev Bras Ortop Engl Ed.

[2] Bederman S.S., Hassan J.M., Shah K.N., Kiester P.D., Bhatia N.N., Zamorano D.P. (2013). Fixation techniques for complex traumatic transverse sacral fractures: a systematic review. Spine.

[3] Matta J.M., Saucedo T. (1989). Internal fixation of pelvic ring fractures. Clin Orthop Relat Res.

[4] Kanakaris N.K., Angoules A.G., Nikolaou V.S., Kontakis G., Giannoudis P.V. (2009). Treatment and outcomes of pelvic malunions and nonunions: a systematic review. Clin Orthop.

[5] Papakostidis C., Kanakaris N.K., Kontakis G., Giannoudis P.V. (2009). Pelvic ring disruptions: treatment modalities and analysis of outcomes. Int Orthop.

[6] Stolberg-Stolberg J., Lodde M.F., Seiß D. (2024). Long-term follow-up after iliosacral screw fixation of unstable pelvic ring fractures. J Clin Med.

[7] Perumal R., S D.C.R., P S.S., Jayaramaraju D., Sen R.K., Trikha V. (2021). Management of pelvic injuries in hemodynamically unstable polytrauma patients - challenges and current updates. J Clin Orthop Trauma.

[8] Gardner M.J., Chip Routt M.L. (2010). The antishock iliosacral screw. J Orthop Trauma.

[9] Khaled S.A., Soliman O., Wahed M.A. (2015). Functional outcome of unstable pelvic ring injuries after iliosacral screw fixation: single versus two screw fixation. Eur J Trauma Emerg Surg.

[10] Iorio J.A., Jakoi A.M., Rehman S. (2015). Percutaneous sacroiliac screw fixation of the posterior pelvic ring. Orthop Clin N Am.

[11] Feinblatt J.S., Phieffer L.S., Lawyer R.B. (2010). Anterior sacroiliac dislocation. Orthopedics.

[12] Page M.J., McKenzie J.E., Bossuyt P.M. (2021). The PRISMA 2020 statement: an updated guideline for reporting systematic reviews. J Clin Epidemiol.

[13] Burns P.B., Rohrich R.J., Chung K.C. (2011). The levels of evidence and their role in evidence-based medicine. Plast Reconstr Surg.

[14] Slim K., Nini E., Forestier D., Kwiatkowski F., Panis Y., Chipponi J. (2003). Methodological index for non-randomized studies (*MINORS*): development and validation of a new instrument: methodological index for non-randomized studies. ANZ J Surg.

[15] Sideri S., Papageorgiou S.N., Eliades T. (2018). Registration in the international prospective register of systematic reviews (PROSPERO) of systematic review protocols was associated with increased review quality. J Clin Epidemiol.

[16] Abumi K., Saita M., Iida T., Kaneda K. (2000). Reduction and fixation of sacroiliac joint dislocation by the combined use of S1 pedicle screws and the Galveston technique: spine.

[17] Mullis B.H., Sagi H.C. (2008). Minimum 1-year follow-up for patients with vertical shear sacroiliac joint dislocations treated with iliosacral screws: does joint ankylosis or anatomic reduction contribute to functional outcome?. J Orthop Trauma.

[18] Sobhan M.R., Abrisham S.M.J., Vakili M., Shirdel S. (2016). Spinopelvic fixation of sacroiliac joint fractures and fracture-dislocations: a clinical 8 Years follow-up study. Arch Bone Jt Surg.

[19] Gauthé R., Lefèvre É., Dujardin F. (2022). Posterior hinge fixation for the treatment of unstable traumatic sacroiliac joint injuries. Orthop Traumatol Surg Res.

[20] Miyake T., Futamura K., Baba T. (2022). A novel technique for stabilising sacroiliac joint dislocation using spinal instrumentation: technical notes and clinical outcomes. Eur J Trauma Emerg Surg.

[21] Leong N.L., Kator J.L., Clemens T.L., James A., Enamoto‐Iwamoto M., Jiang J. (2020). Tendon and ligament healing and current approaches to tendon and ligament regeneration. J Orthop Res.

[22] Cheng C., Shoback D. (2019). Mechanisms underlying normal fracture healing and risk factors for delayed healing. Curr Osteoporos Rep.

[23] Banierink H., Ten Duis K., Wendt K., Heineman E., Ijpma F., Reininga I. (2020). Patient-reported physical functioning and quality of life after pelvic ring injury: a systematic review of the literature. Farouk O, curatore. PLoS One.

[24] Kim C.H., Kim J.W. (2020). Plate versus sacroiliac screw fixation for treating posterior pelvic ring fracture: a Systematic review and meta-analysis. Injury.

[25] Banaszek D., Starr A.J., Lefaivre K.A. (2019). Technical considerations and fluoroscopy in percutaneous fixation of the pelvis and acetabulum. J Am Acad Orthop Surg.

[26] Pollard T.G., DeLeon J.C., Parry J.A. (2023). Tips and tricks for the reduction and fixation of sacroiliac joint fracture-dislocations. J Clin Orthop Trauma.

[27] Elzohairy M.M., Salama A.M. (2017). Open reduction internal fixation versus percutaneous iliosacral screw fixation for unstable posterior pelvic ring disruptions. Orthop Traumatol Surg Res.

[28] Katsoulis E., Giannoudis P.V. (2006). Impact of timing of pelvic fixation on functional outcome. Injury.

[29] Jacob A.L., Messmer P., Stock K.W. (1997). Posterior pelvic ring fractures: closed reduction and percutaneous CT-guided sacroiliac screw fixation. Cardiovasc Intervent Radiol.

[30] Wang Y., Du X., Tomaszewski R., Journeau P., Mayr J. (2024). Operative management of sacroiliac joint dislocation in children with unstable pelvic fractures – a STROBE-compliant investigation. J Orthop.

[31] Zhou W., Chen J., Pei X. (2023). Incidence of and risk factors for screw loosening after iliosacral screw fixation for posterior pelvic ring injury. Orthop Surg.

[32] Ziran N., Collinge C.A., Smith W., Matta J.M. (2022). Trans-sacral screw fixation of posterior pelvic ring injuries: review and expert opinion. Patient Saf Surg.

[33] de Ridder V.A., Whiting P.S., Balogh Z.J., Mir H.R., Schultz B.J., Routt M.C. (2023). Pelvic ring injuries: recent advances in diagnosis and treatment. OTA Int.

[34] Hoffmann E., Lenoir T., Morel E., Levassor N., Rillardon L., Guigui P. (2008). Posterior bridging osteosynthesis for traumatic sacroiliac joint dislocation: a report of seven cases. Eur J Orthop Surg Traumatol.

[35] Koshimune K., Ito Y., Sugimoto Y. (2016). Minimally invasive spinopelvic fixation for unstable bilateral sacral fractures. Clin Spine Surg Spine Publ..

[36] Sagi H.C. (2009). Technical aspects and recommended treatment algorithms in triangular osteosynthesis and spinopelvic fixation for vertical shear transforaminal sacral fractures. J Orthop Trauma.

[37] Dujardin F.H., Hossenbaccus M., Duparc F., Biga N., Thomine J.M. (1998). Long-term functional prognosis of posterior injuries in high-energy pelvic disruption. J Orthop Trauma.

[38] Tornetta P., Matta J.M. (1996). Outcome of operatively treated unstable posterior pelvic ring disruptions. Clin Orthop.

[39] Tile M. (1988). Pelvic ring fractures: should they be fixed?. J Bone Joint Surg Br..

